# Ethnoecology - The best medicine against allergy?

**DOI:** 10.1186/s13002-015-0013-7

**Published:** 2015-05-02

**Authors:** Zsolt Molnár

**Affiliations:** MTA Centre for Ecological Research Institute of Ecology and Botany, Alkotmány u. 2-4., H-2163 Vácrátót, Hungary

**Keywords:** Landscape, Nature conservation, Pasturing, Steppes, Traditional ecological knowledge

## Abstract

This essay, which is the 6^th^ in the series “Recollections, Reflections, and Revelations: Ethnobiologists and Their First Time in the Field”, is a personal reflection by the researcher on his first field experiences with ethnobiology. Author writes on how Hungarian herders in the Hortobágy salt steppes and Csángó people in the Carpathian mountains changed his views on landscape, vegetation, local people, traditional small-scale grassland management and finally, ecology and nature conservation.

## Introduction

A botanist usually spends each summer working in the forests and meadows. Since 1997, I was not able to carry out this sort of work during the months of August and September, when the highly allergic alien ragweeeds (*Ambrosia artemisiifolia*) were blooming. As it proved later, this was one of the reasons why I am now working in the field of ethnoecology.

I was born into a lucky family. My dad is a forester, a violaist and long-term performer in a symphony orchestra. My mum is a teacher of song, music and mathematics, and has always been seriously interested in everything ethnographic. I spent my childhood in a vibrant and holistic atmosphere: trips to the forest at the weekends, classical concerts, art exhibitions, and lots of reading in the evenings, travelling around the country in the summers (various landscapes, museums, ethnographic open air museums). I also played the piano and double bass. We had a small garden where we grew fruit and vegetables. My pastime was birdwatching, and later plant identification, but I also very much liked books on history and ethnography, particularly on the life of the fishermen of the former vast marshes. The old maps created a separate world to me. I was constantly ‘all ears’ whenever my father explained with great enthusiasm what could be seen on these yellowed sheets of paper. Other times we searched for long-discarded items in the hidden corners of farmhouse backyards, all of which would assume new functions in our home. A disposed clay pot that we discovered once soon became a holder for drawing pencils.

### Becoming a scientist

By the end of the high-school years, I had decided to become an ecologist. Thus, I left behind my beloved little town surrounded by mountains, and set up residence in a city of the unusually flat Hungarian Great Plain. Here I met my future wife, Mariann, who while keenly interested in history, literature, and art, had finally chosen to pursue a career in biology. Though we were both ecology major students, we had to study molecular biology almost exclusively because this, or so we were taught, would inevitably change the world during the 21^th^ century. Instead, I found myself frequenting a distant and mysterious place, the empty *puszta* (salt steppe), where I made friends with local peasants and herdsmen. We found a shelter from the hot sun behind the wooden wall of a draw-well where they would tell the tales of the old *puszta*, about how it was grazed in the past, and how that *puszta* became the *puszta* of today, serving as home to so many plants and birds.

It was here, in this *puszta*, that I met my first interviewee, whom I would come to know as Uncle Feri. He lived in an old worn-out mobile home. Although many of the villagers rejected him, over a period of time, I was able to befriend him. He was quite reserved, and at first, we exchanged words only about the daily news. As he began to see my deep interest in the past and the peasant way of life, with the aid of a bottle of *pálinka* (brandy), we soon became like grandfather and grandson. His former farmhouse was demolished during the communist period and then he was jailed, because he refused to join the socialist cooperative. After he was freed, he never built another home. He lived for a long time in a pit-house that nobody could take from him. It was only after several long years that he moved into this “luxurious villa”, where he received me so many times. It kept us warm in winter, and cool in summer. At this time he began to talk, about the *puszta* and its people, about the construction of draining ditches, and about ploughing the grassland. Unfortunately, by now, the shabby mobile home has perished. Memories of Uncle Feri accompany me on my collecting trips. What I learnt from him, his poise and dignity, I shall never forget. At that time I was not totally aware why the strange world of Uncle Feri affected me so deeply, why my recollections of a socially marginalised person of the *puszta* became so valuable and relevant to me. It might almost have been a sacred initiation.

Later, in the huge library of the university I had a chance to read lots of historical and ethnographic works. Later, already a research fellow of the Hungarian Academy of Sciences, I searched through museum archives and manuscript collections for information on the history of the Hungarian salt steppes. During a trip to England as a visiting fellow (Tempus), I became acquainted with several British landscape historians and their works e.g. [1], but I was mostly impressed by a book entitled *Countryside Heritage* [2] that applied a holistic approach to the presentation of landscape history and contained rich photographic illustrations. My conviction of the need for historical ecological research was aroused. I was eager to write a similar book on the Hungarian Great Plain.

**Figure 1:**
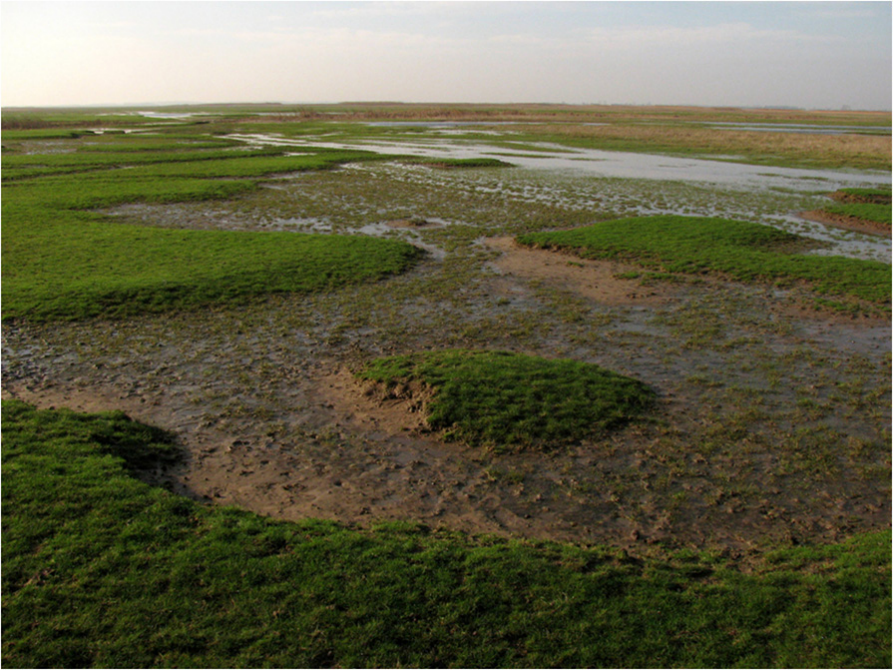
One of the most ancient landscapes in Hungary: the salt steppes of the Hortobágy (Photo by Zsolt Molnár).

At that time (at the beginning of the 1990s), it was not customary for a Hungarian ecologist to show interest in historical and ethnographical questions, nor even to spend a good part of his time reading about them. Even my supervisor asked me: “Is this your hobby, Zsolt”? A young fellow researcher of mine, however, concluded that I had somehow been transformed from a botanist into some kind of historian, but because I was able to interpret botanical phenomena using novel ecological methods, increasingly my colleagues began to accept this new, so-called landscape historical approach. By now, this approach has become widely used in our country. During my work, I also had to turn on their heads and reject old beliefs and dogmas relating to the Hungarian landscape. This new approach helped me show that the commonest grassland type in Hungary, the salt steppe, was not anthropogenic in origin and that it did not develop secondarily following the introduction of river regulations during the 19th century (as claimed by the accepted scientific model), because the countryside looked just about the same before then. We were thus able to reinterpret this ‘so-called’ anthropogenic landscape as one of the most ancient types to occur in Hungary [3], Figure 1. Later we also demonstrated the reason that the forest understories on the Hungarian Great Plain are so poor in forest specialist species is because they were substantially over-utilized in the past, or because many of them had developed secondarily [4]. My wife and I unearthed yellowed sheets of old maps and began to reconstruct the vegetation patterns of past ages [5].

### Family, Hungarian traditions, and ecology

Our children were born in 1991 and 1993. When raising them, we tried hard to make use of Hungarian folk traditions (games, tales, celebrations, everyday sacrality). Although my wife and I had already brought with us the need for traditions from our childhood homes, by chance, we even had an opportunity to study Hungarian traditions for a period of two years at a folk-highschool. During lectures and lecture breaks, field practicals and summer camps, we met many fascinating people, but we were also astounded at how little teachers educated in the humanities knew about ecology and how limited in its scope was their world view.

In order to address this deficiency, we, together with an enthusiastic team, organized a series of folk-highschool lectures entitled ‘Landscape and Man’. We invited lecturers every two months who told us about the unity of Hungarian culture and landscape. We met genuinely inter-, multi- and even transdisciplinary approaches e.g. [6-8]. This folk-highschool also played an another important role since, by now, about a dozen young and middle-aged, ecologists working in Hungary, were able to apply the holistic approach (biological, historical, ethnographical) to their research. It was during these years that I in fact became a fully-fledged ethnobiologist, although I was still not aware of this at the time.

### Suspended summer field work due to ragweed allergy

Although my parents raised me with utmost care, I was often ill, and had to take lots of medications in my childhood. This was perhaps the reason for my allergy. At this time (in the late 1990s), I was working in the middle of the Hungarian Great Plain and, since my allergy meant that I often lost about one-third of the time available to me to undertake seasonal field work, I decided that I should search for a place in the Carpathian Basin, where my misery was not so pronounced. Since, by disposition, I was not able to tolerate long periods indoors, we began to schedule our summer family holidays to coincide with the allergy period and the further we were from ragweed, the better. As a result of this, we eventually ended up in the eastern part of the Carpathian basin, in Transsylvania, which is inhabited by Hungarians. The fresh (ragweed-free) air of its hilly land was good, and we could spend more and more time among its friendly people, called Székelys. From this time on, our holidays were gradually transformed into something indefinable, a combination of partly professional and partly familial activities filled with joy and challenges for us all, and in which local people increasingly became participants and players.

I remember, for example, the dawn when we joined a genuine goat herd in a tiny village of the Bihor Mountains. Béla daily gathered 170 goats from the village. Firstly, we drove them up to the mountain top, where a birch grove needed to be grazed, and then a rose-hawthorn thicket needed to be “controlled”. At noon, like the herdsman, we too took a nap to the quiet, but monotonous sound of sheep-bells. In the afternoon, we first “took care” of a coppice that grew in the clearing of a beech wood, then we struck some delicious fruit off some wild apple and pear trees with a pole and offered them to the goats as a dessert or simply as “payment” for a good day’s work. By evening, I realized that almost no grasses (but mostly shrubs and trees) had been grazed by the goats. I was astounded that grazing the animals was not quite what I had expected it to be. To put it more bravely, humans certainly have an impact on the vegetation, but not in the way that I had learnt to date from ecology books or heard at conferences. For instance, we did not know before this – and it certainly had not been taught us at universities that sheep can graze even in forests. This used to be mentioned only with regard to pigs. I have even heard from Béla that sheep prefer beech masts to oak acorns.

Several years and holidays have passed since I became aware of the enormous amount of ecological knowledge that people living in this part of the world possess. As it was our summer holiday, I never took a plant identification guide with me on these Transsylvanian trips. However, as a researcher of the plains, I hardly knew anything about mountain plants. In the end, I could no longer surpress the botanist inside me and so it was that when we next met someone in the field, I asked him the names of all the plants, though not yet for any particular purpose. I gradually realized what a large number of plant species these people know, and started systematically to put on paper the names that the local people used for them. When it became possible, we joined them as they grazed their herds, helped them cut and turn-over the hay, and stockpiled it into haystacks. We essentially began to collect data and conduct participatory fieldwork during these so-called family holidays. We got involved in the life of the local people, because they liked it when we were all together, but the work could no longer be put aside, so we went at it collectively. As a consequence, our children had learnt, by the age of 11–12, how to make hay, how to drive horses, how to feed animals, and were very pleased to accompany anyone as they harvested the fruits, mushrooms and medicinal herbs of the forest.

Hungarian folk music culture is very rich. In the rather isolated and least prosperous parts of Transsylvania, musical traditions still survive today. After ten of my childhood years playing the piano, I began to play the traditional flute in the folk highschool. What an experience it was! We were learning from an ethnomusicologist, Zoltán Juhász [9]. We stared at his fingers as we learned. Instead of reading music sheets, we played by ear, and instead of playing at concerts, we played to each other. We also sang a lot. I learnt several hundreds folk songs within a few years. I visited Transsylvanian flute players (who play the flute as part of their tradition, not having learnt it at school), one after the other, at their homes so as to enable personal contact help me understand their music even more deeply. I realized how traditional knowledge and its transfer differs from that of scienfific knowledge. While sitting on the moutain top and playing the flute, I eventually made the decision to start studying traditional ecological knowledge.

In one of the flute camps, we were singing and playing the flute around the campfire, when, by chance, I managed to get a seat right next to András Berecz, who is a well known folk musician. He is a pioneer of studying local musicians’ perception of song techniques and also an eminent story-teller of folk tales [10]. As I already suspected that traditional knowledge had become extinct in the Hungarian Great Plain, I asked him how I should begin to study it. He answered as though we were part of a folk tale: “Go to the east! When you return to our little homeland, you will notice the remaining crumbs.” Off I went to try my fortune.

### Gyimes, the appealing place – the first consciously chosen ethnobiological field

**Figure 2:**
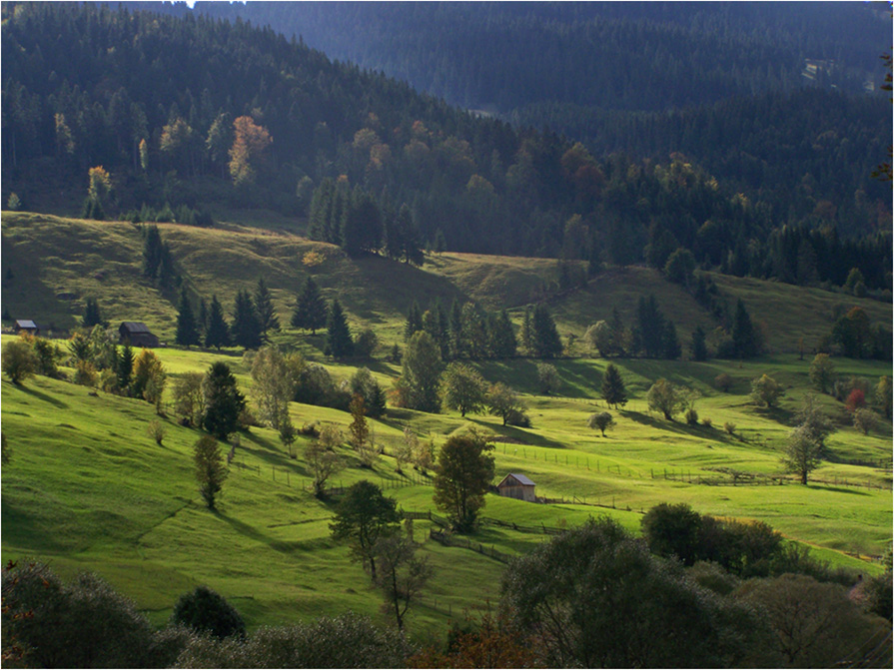
The beauty of the Gyimes mountains (Photo by Dániel Babai).

One of the most easterly regions inhabited by Hungarians is Gyimes. This area is very famous, or rather, fashionable, because of its rich and well-preserved traditions. It lies amongst the mountain ranges of the Carpathians, close to the thousand-year-old border between Hungary and Romania (today it is part of Romania). Although I had never been there before, I has stubbornly thought that no matter where I would do my research, it would not be the Gyimes. My wife’s curiosity, however, was stronger than mine. She devised cunning plans just to get me there. For a day, she begged and at last, I gave in. We went, but were not able to make closer contact with one of the local inhabitants. Even so, I secretly fell in love with the place (Figure 2). So, the following year, we set out for the second time, again just for a day under the pretence of letting my wife show Gyimes to her parents. My father-in-law asked the very first person whom he could talk to whether there was anyone there who could play the flute. The answer was “but of course, my husband”. A friendship that has lasted until the present time soon ensued, and I was seduced. He pulled out his 100-year-old (in fact, older) flute and began to play. From the very first moment, his family was very friendly, and even invited us to spend a week with them in the winter. So we returned - in February. There was a hard frost and there was snow everywhere. All day we played the flute, cut firewood, and made cheese. Unlike most Hungarian families, people in Gyimes were too proud to send their daughters as servant-girls to the cities, and thus their farming, vegetable garden, food and furnishings were much less influenced by the urban lifestyle. As they can hardly use the land for agriculture owing to the mountain climate (they produce only small amounts of potatos), their subsistence is based on the biomass of meadows and forests (milk, cheese, veal, wood). It was an elementary experience to bake bread in a kiln, weave wool, play the flute in the evenings, and transport wood on a horse-drawn sleigh. In the summer, we showed up there again, but this time, with our children.

We listened with longing in our hearts to our acquaintees when they told us about the alpine pastures and the small huts called *esztena*. Some years passed, and we joined them on their way to the *esztena* to take our turn in the *szer* (the supervision of grazing animals, divided into week-long periods shared between relatives). In the morning, I was given a scythe, and I joined the others at mowing. My bent back is clearly visible in the picture taken at that time. When we scythed a species-rich alpine meadow, entire bouquets of flowers fell to the ground at every slash, among which there were many plants that are protected by nature conservation back home (i.e. *Trollius, Eriophorum, Gentiana, Dactylorhiza, Ophioglossum*). This was the first time for me to feel that conservation work was also fodder-yielding work. From that time, I have had a different attitude towards writing a conservation management plan.

But still I had not taken a plant identification key with me. Neither did I read books on ethnobiology. I thought that maybe, by the time I had finished writing up my doctoral dissertation on the landscape history of the Great Plain, which would take years, an exciting research topic would have arisen. I waited and listened, scythed and helped with the fieldwork. However, I also took increasingly more detailed notes on the folk names of plants and the statements and stories relating to the vegetation. It seemed that almost all plants had their own local name (we now know that ‘only’ about 260 out of some 450 species do).

**Figure 3:**
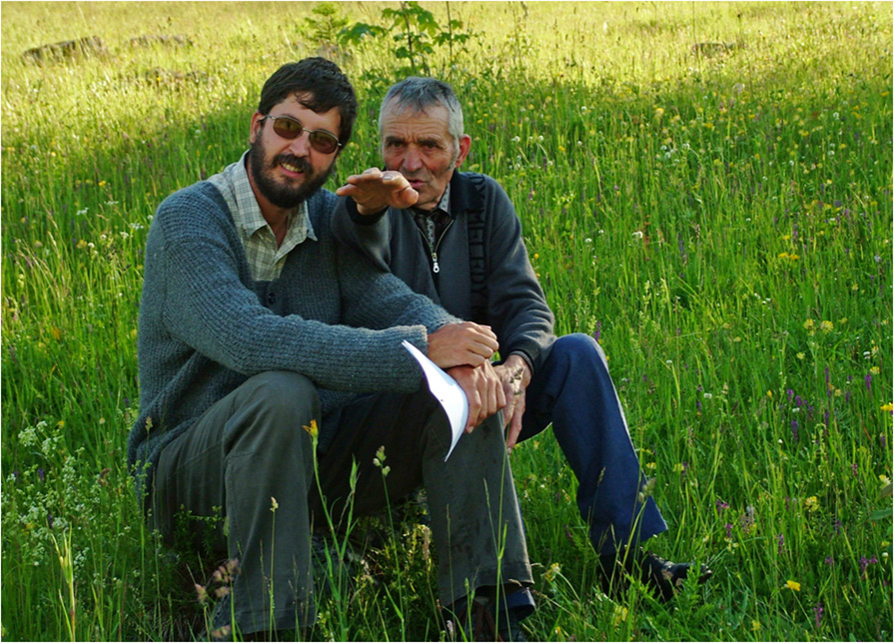
Gyimes (Eastern Carpathians), our first real ethnoecological study site (Károly Prezsmer explaining meadow management) (Photo by Ábel Molnár).

During 2005, my student (Dániel Babai) and I decided to start a research project. He simultaneously took courses in biology and ethnography. Firstly, we wanted to find out what the people of Gyimes know about the ecological requirements of different plant species. By now, we had already asked about 30 people the same question about 4500 times, namely, In what kind of place does this plant like growing? It turned out that the Gyimes people are able to distinguish more than 140 different kinds of habitats [11]. Later, we also learnt that they know a great deal about vegetation dynamics and the effect of grassland management on vegetation [12] (Figure 3). We gradually realized that traditional ecological knowledge is not just interesting and important in itself, but is also necessary for vegetation science and nature conservation. We began a literature search. As it turned out, we could rely on the great work of our predecessors. Attila Szabó T., János Péntek and János Rab [13,14] had conducted ethnobotanical studies in the nearby Gyergyó and the more distant Kalotaszeg regions decades ago. They collected folk plant names, information on the use of plants, and selected names related to vegetation from toponyms. However, they had not studied folk taxonomy and the living knowledge of vegetation and vegetation dynamics. Having reviewed the international literature, it was clear that not many people had studied nor currently study traditional ecological knowledge related to vegetation and small-scale meadow management. What is more, in the case of most interviews, the mother tongues of the interviewees and researchers are different, and therefore the opportunity of gleaning much useful information is often lost. We also started learning anthropological methods, and improved our interviewing techniques. One of our most important early readings was a book by Fikret Berkes [15]. Now we see the benefit of waiting and listening for many years.

### Searching for “crumbs”: herdsmen’ knowledge of vegetation and traditional grazing in the Hortobágy salt steppe

After my return, I aimed to study the ethnobiology of the salt steppes, my favorite type of landscape. I already knew it very well, having studied its current vegetation and landscape history for long. Since 1985, I had talked to many herdsmen, sometimes even asking for plant names. Sadly, I could not get more than 4–5 names, except in one case. A colleague of mine, working for nature conservation, also asserted that herdsmen name only very few plants, and hardly know any grass species. Today, I know for certain that the typical botanist’s approach is not sufficient to access traditional ecological knowledge. One needs to know the appropriate methods, without which we may easily take the wrong path or scratch only the surface.

The herdsman who influenced me the most during the first years was István Gojdár, whom I met in 1995. He was not born to a herdsman family. He evolved from being a typical rural person to becoming a herdsman. He could listen very keenly, and particularly loved the scent of the *puszta*. Once, he even wrote a letter to me about it. It was the third letter of his entire life: “At the time when the *tippan* (*Festuca pseudovina*) throws out its head, and when the dew settles out in the evening breeze, which then ruffles the *puszta*, and then brings the blend of those millions of odours to me, no deodorant on Earth can surpass it. This is a different world.” He could list the names of 70 plants. Even spikerushes (*Eleocharis*) and alkaligrass (*Puccinellia*) had unique names.

**Figure 4:**
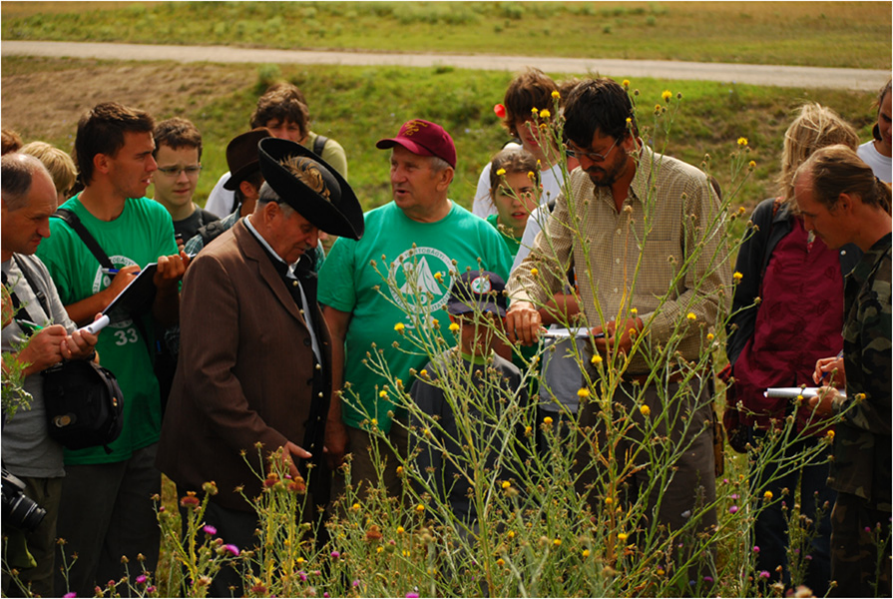
One of our first herder informants (István Nagy) who teaches plants to the participants of a summer research camp at the Hortobágy (Photo by Ábel Molnár).

I started picking up the “crumbs” in the Hortobágy (the largest salt steppe in Central Europe) in 2008. It was quite unexpectedly that Dániel Babai found a long-forgotten series of articles published 60 years ago by the forester Béla Tikos in a linguistic journal [16]. Armed with these papers, we set out to do research, and even on the first day we collected 55 plant names from a single cattle herder. These were supplemented with another 25 names on the next day in the field (Figure 4). The many “crumbs” filled me with enthusiasm. From the autumn, Károly Hoffmann, a student of mine, and I began to systematically visit herdsmen. After just five days and only the tenth herdsmen, we could close the papers of Tikos. We collected the names of more plants (although he emphasized that if he did not collect and publish those names, they would disappear forever. Indeed, some of the names have been forgotten since then, but only few.) It was an astonishing experience to learn that this knowledge is still alive, and that not only “crumbs”, but entire “slices” can be recovered. Since then, we have talked to 92 herdsmen for 2–3 (or more) hours, and spent lots of time with them in the *puszta*. We obtained more than 500 folk names of about 243 species, that is about 162 folk taxa. Although the salt steppe is a much simpler landscape than that of Gyimes, we could collect more than 180 folk names for the ca. 40 habitat types, and we even learnt a lot about vegetation dynamics from our herdsmen friends [17,18].

We learnt, perhaps, the most from old lone herders. They have time, and often, if we also have time, we can talk for many hours. We walked out to the *puszta* with them many times, where they patiently shared their knowledge about plants and herding both in old and recent times. It happened that twice during such springtime walks, they identified similar green tufts of grasses to species level (*Poa bulbosa, Festuca pseudovina*, *Hordeum hystrix, Lolium perenne, Bromus hordeaceus*). Do they not know grasses? So far, we have found a total of 29 folk taxa that refer to grasses, sedges and similar monocots.

**Figure 5:**
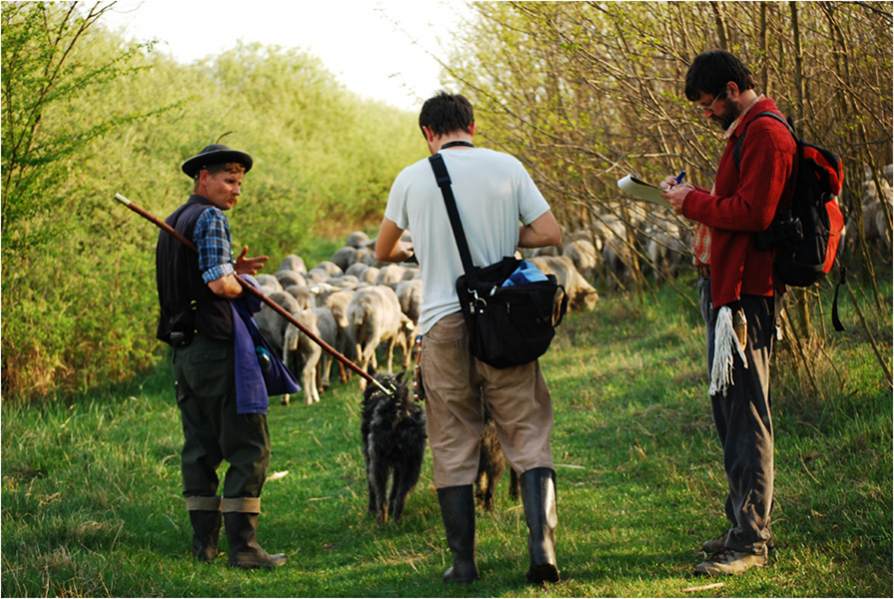
Learning from a shepherd during grazing (László Sáfián, Hajdúsámson, Hungary) (Photo by Ábel Molnár).

Later, we joined them during pasturing. Since 2009, we have spent more than 200 days with them, and learnt about the many types of decisions a herder has to make in order to adapt to the spatially and temporally heterogeneous fodder availability of his pasture [19], Figure 5. To get up at 3 a.m. and graze the livestock until 11 p.m. (with some rest around noon) helps you understand and respect the knowledge of these herders.

As the area is part of a national park, our experiences can be directly applied to nature conservation. For example, one of the greatest conflicts in the national park is that meadows are allowed to be mowed only when the bird nesting season is over (from the 15th of June or 1st of July). Steppe grasses have dried out by this time, and the value of the hay drops to its lowest. Herdsmen do not understand why a bird is worth more than a sheep. Grass phenology is so important that it is reflected in the local folk names. We learnt that the name of the dominant grass in the meadows (*Alopecurus pratensis*) is different in the spring and autumn (when green) and in the summer when it is yellow. It is *perje* when green, which in Hungarian is given to green and tasty fodder plants (see the related words of *paréj, paraj, burján*), whereas it is *pipaszurkáló* (literally pipe-cleaner) when yellow, and hard and inedible. The national park lets the grass be cut when the latter name is applied to the plant. EU subsidies may make up for lost profits, but the feeling of loss these herders have cannot be taken away from them. We hope that our studies will help resolve these and similar conflicts between herders and conservationists.

Sadly, our research is still not valued very highly by some ecologists, in much the same way that our landscape historical research was not appreciated twenty years ago. When are you going to do real ecology again? – asked one of my colleagues. I hope that just as in the case of landscape history, this type of question will no longer be asked in a few years. It is clear that without this and similar local knowledge, ecology will remain divorced from society. We argue that ecology will be less able to contribute to nature conservation if it ignores rural people and their detailed understanding of their environment [20]. The latest international programme, the Intergovernmental Science-Policy Platform for Biodiversity and Ecosystem Services (IPBES) [21] also calls for deeper dialoge between knowledge systems. Traditional ecological knowledge is one of these.

Furthermore ethnobiological research also amply privately rewards the individual ethnobiologist. One may collect useful ideas for running a family or raising kids, for working in the kitchen or curing illnesses, but learning about the life of our interviewees (better still, our teachers and often friends) may also yield lots of ‘useful’ lessons. Our children have also learnt a lot. Their hobbies are strongly associated with their experiences during our summer holidays. My son, Ábel, visits herdsmen to take photographs, and learn about traditional grassland management, and my daugther, Kata, collects and raises medicinal herbs that she uses for making tea mixtures and remedies. Above all, ethnobiological research is great fun. Why don’t you just do it?
